# BYPASS-OMA: Hypoglycemic Hyperinsulinemic Nesidioblastosis after Gastric Bypass Surgery—A Case Report and Review of the Literature

**DOI:** 10.1155/2022/5472304

**Published:** 2022-12-22

**Authors:** Jessica Cao, Cindy Kim, Thatcher Huynh, Amanda Frugoli, Heidi Henson, Vera Valdez, Tricia Westhoff-Pankratz

**Affiliations:** ^1^Western University of Health Sciences, Community Memorial Hospital, Department of Internal Medicine, 147 N Brent St, Ventura, California 93003, USA; ^2^Community Memorial Hospital, Department of Graduate Medical Education, Department of Internal Medicine, 147 N Brent St, Ventura, California 93003, USA; ^3^Community Memorial Hospital, Department of Internal Medicine, Department of Endocrinology, 147 N Brent St, California 93003, USA

## Abstract

This rare case vignette describes hypoglycemic, hyperinsulinemic nesidioblastosis in a female patient with prior Roux-en-Y gastric bypass. The patient presented with severe symptomatic hypoglycemia resistant to IV dextrose and diazoxide, requiring surgical resection. Traditional imaging found nonspecific findings, and biochemical analysis was inconsistent with insulinoma. A gallium-68 dotatate PET scan was utilized to successfully localize the tumor in the distal pancreas. She underwent laparoscopic resection of the distal pancreatic lesion with resolution of her symptoms and return to euglycemia. The histological evaluation confirmed the diagnosis of nesidioblastosis. Nesidioblastosis is a rare complication of bariatric surgery that may be more clinically relevant with rising prevalence of obesity. Diagnosis with conventional imaging modalities may be challenging; however, the dotatate PET scan may have high utility in detecting lesions. It is essential for clinicians to consider nesidioblastosis in the differential diagnosis of hyperinsulinemic hypoglycemic conditions and recognize there may be a link with increasing rates of bariatric surgery.

## 1. Introduction

Hyperinsulinemic hypoglycemia (HH) refers to uncontrolled insulin release from neoplastic beta cells or functionally defective pancreatic beta cells that results in hypoglycemia. This broad term is used to describe a number of disease pathologies with similar mechanisms and clinical presentation. Adult-onset, hyperinsulinemic hypoglycemic syndromes have a wide differential that usually necessitates systematic evaluation. Disease syndromes from endogenous insulin production include insulinoma, nesidioblastosis, and dumping syndrome. The remaining syndromes include surreptitious sulfonylurea or insulin use which needs a biochemical analysis for differentiation.

Although rare, insulinomas are the most common cause of hyperinsulinemic hypoglycemia [1]. Insulinomas represent a type of pancreatic neuroendocrine tumor (pNET) or islet cell tumors. They are usually solitary and benign tumors of the pancreas. They are difficult to distinguish from nesidioblastosis as both disorders result from abnormal pancreatic beta cell function. The ultimate distinction relies on histopathologic evaluation [2]. Without biochemical analysis, insulinoma and factitious hypoglycemia from surreptitious sulfonylurea or insulin use can be indistinguishable. Surreptitious insulin use will show increased insulin with decreased C-peptide levels during the hypoglycemic episode due to the negative feedback imposed by exogenous insulin [3]. The overuse of insulin secretagogue medications such as sulfonylurea may mimic an endogenous insulinoma by stimulating the endogenous release of insulin from pancreatic beta cells and increasing insulin, proinsulin, and C-peptide levels [3]. For this reason, sulfonylurea screens have been developed. Postprandial hypoglycemia or reactive hypoglycemia can be a clinical mimicker for insulinoma or nesidioblastosis but has a distinct pattern with food intake and usually occurs within four hours after eating. Postprandial hypoglycemia can be seen in otherwise healthy patients after eating high-carbohydrate meals. It is most often seen after gastric bypass surgery but can occur after esophagectomy, fundoplication, sleeve, or total gastrectomy. Dumping syndrome was coined to describe the increased amounts of undigested food being rapidly transmitted to the small intestine resulting in a myriad of neuroglycopenia symptoms. This occurs more commonly in patients with gastric bypass surgery due to reduced foregut length [4]. The pathophysiology suggests an increase in neuropeptide release driven by increased incretin secretion stimulating beta cells' insulin production with resulting exaggerated or hyperinsulinemic response [4, 5].

Nesidioblastosis is likely to be an increasing etiology for HH conditions, with rising bariatric rates. Nesidioblastosis was first identified in 1938 by Dr. Laidlaw who described the diffuse proliferation of pancreatic duct epithelium [6]. Over time, the definition has evolved to include a congenital or acquired abnormal function of pancreatic beta cells that results in persistent hypoglycemia that is not explained by insulinoma [7]. In newborns and hyperinsulinemic hypoglycemia of infancy, the underlying pathophysiology is suspected to be sporadic or congenital genetic mutations. These genetic mutations result in either focal or diffuse islet cell changes [8–10]. This pathogenesis is hypothesized to be different for adult onset. The condition is rare for both infants and adults. The prevalence has not been well studied in adults but is the etiology in approximately 0.5–5% of cases of hyperinsulinemia [1].

The acquired form of nesidioblastosis most often occurs following an Roux-en-Y bypass, and pathophysiology remains controversial [8]. Various hypotheses have been proposed, including hindgut theory, foregut theory, pre-surgical obesity theory, vagus nerve theory, elevated serum bile acid theory, dumping syndrome theory, and other undiscovered pathways. Foregut theory proposes that unidentified anti-incretins released from the foregut are downregulated in bypass surgery. This increases incretin release and stimulates a decrease in blood glucose levels [11]. Hindgut theory proposes that nutrients bypass the foregut, reach the hindgut, and lead to the release of glucagon-like peptide (GLP-1) and peptide YY [11]. After gastric bypass, GLP-1 has been shown to increase postprandially, which is thought to increase the pancreatic *β*-cell mass [12, 13]. In one study by Service et al., histopathology was compared and nesidioblastosis in obese gastric bypass patients had histological islet cell hypertrophy compared to those in obese patient controls without gastric bypass [14]. The presurgical obesity theory proposes that nesidioblastosis occurs from continued *β*-cell hypertrophy in response to presurgical obesity [11]. Meire et al. reexamined the six specimens from the study of Service et al. and concluded that patients with post-bypass hypoglycemia did not have an increased *β*-cell area compared to that of obese controls [14, 15]. During bypass surgery, manipulation of the vagus nerve may suppress ghrelin release, decreasing hunger and increasing insulin secretion [11]. This may exacerbate the propensity for hypoglycemia [14]. Although not fully understood, elevated serum bile acids after surgery have effects similar to bile sequestrants, impacting gluconeogenesis and GLP-1 secretion [11]. It is also possible that there are undiscovered gastrointestinal hormones and pathways, representing a new area of research [11].

It is essential to recognize that hypoglycemia after gastric bypass surgery does not strictly indicate altered pathology but is a side effect of altered anatomy and function. Meier and colleagues examined histopathology from 53 specimens, comparing patients with prior gastric bypass to obese patients and lean controls from autopsies [15]. Not surprisingly, patients with postprandial hypoglycemia after gastric bypass did not have increased beta cells. This is consistent with the most common postprandial hypoglycemia in gastric bypass patients or dumping syndrome. In contrast, cases of insulinoma and nesidioblastosis represent inappropriately increased insulin secretion with associated acquired pathology of an increase in beta cells. These diagnoses are based on biochemical analyses and supporting histopathology.

In this case vignette, we describe a rare hypoglycemic hyperinsulinemic nesidioblastosis in a 37-year-old female patient with prior gastric bypass and review the relevant diagnostic evaluation. As our population continues to have an increased prevalence of obesity, it is also likely that there will be an increase in rates of bariatric surgery and nesidioblastosis [13]. We present this rare case to raise awareness among physicians.

## 2. Case Presentation

A 37-year-old woman with a history of hypothyroidism, remote amphetamine use, and gastric bypass surgery presented with neuroglycemic episodes. She had gastric bypass surgery with Roux-en-Y 10 years ago and initially lost 120 pounds. In the last four months, she has had recurring bouts of syncope, diaphoresis, and unintentional weight loss of 60 pounds. She reported a history of hypoglycemia and stated she was told a pancreatic mass was found in her previous hospitalization four months earlier. She had no history of diabetes but had self-measured blood glucose levels of less than 50 mg/dL despite using over-the-counter glucose tablets. At the time of her presentation, she had taken up to six glucose tablets daily for low blood sugar. She had severe hypoglycemia of 43 mg/dL, non-anion gap metabolic acidosis, and hypokalemia at 2.8 mmol/L. She required ICU level of care for hourly blood glucose monitoring. A 3-day fast was attempted but her persistent and symptomatic hypoglycemia necessitated dextrose infusion, and her fasting was discontinued before the end of the first day. Diazoxide and continuous intravenous dextrose 5% in water (D5W) were given to attempt to normalize her blood glucose but with poor response to treatment and persistent hypoglycemia.

An endocrinology consult was obtained and a systematic biochemical evaluation was initiated (Table 1). Her blood glucose at the time of the studies was less than 50 mg/dL. Her fasting insulin, C-peptide, and proinsulin were inappropriately normal. Despite her hypoglycemia, the C-peptide and insulin levels were not suppressed as would be expected with normal physiology. Other differentials indicative of sulfonylurea use, autoimmune diabetes, defective cortisol secretion, neuroendocrine tumors, and celiac disease were excluded (Table 2). On hospital day 10, her glucose continued to be low at 47 mg/dL despite diazoxide use. C-peptide and fasting insulin levels at 3.1 ng/mL (reference range: 0.8–3.5 ng/mL) and 3.6 (reference range: 2.0–19.6 ulU/mL) were not suppressed despite severe hypoglycemia (Table 1). Total parenteral nutrition (TPN) was started on her with D20% concentration with amino acids to help manage her hypoglycemia.

**Figure 1:**
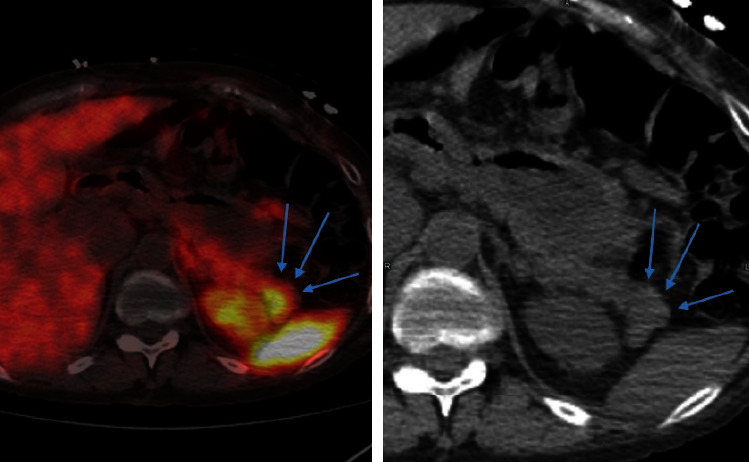
Gallium-68 dotatate PET scan and corresponding abdominal CT. Gallium-68 dotatate PET demonstrates a focal area of radiotracer concentration in the distal pancreas consistent with a neuroendocrine tumor in the tail of the pancreas. Compared to CT imaging of the distal pancreas, there is a contour abnormality near the tail, suggesting a possible lesion.

She had multiple imaging studies, including abdominal MRI and CT abdomen and pelvis, which had no significant findings except for a contour abnormality near the pancreatic tail identified by MRI imaging (Figure 1). She then underwent endoscopic ultrasound by gastroenterology to assess the pancreas, which was reported as normal. Further testing using a gallium-68 dotatate PET scan demonstrated a focal area of radiotracer concentration in the distal pancreas, possibly consistent with a neuroendocrine tumor (Figure 1).

**Figure 2:**
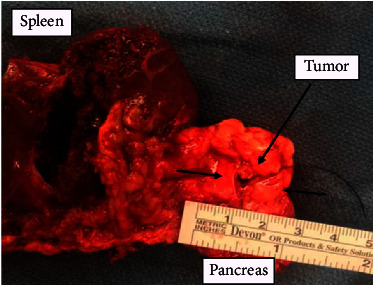
Gross pathology demonstrating spleen and pancreas with a 0.6 × 0.6 × 0.3 cm pancreatic mass.

**Figure 3:**
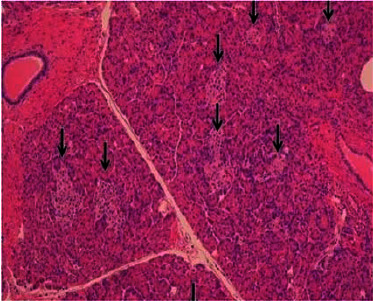
Immunohistochemical staining for chromogranin-highlighted expanded islet cell nests with diffuse expansion of islets of Langerhans.

**Figure 4:**
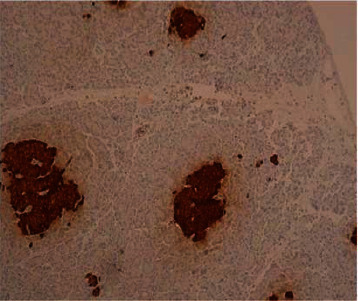
Immunohistochemical staining for synaptophysin-highlighted expanded islet cell nests.

**Figure 5:**
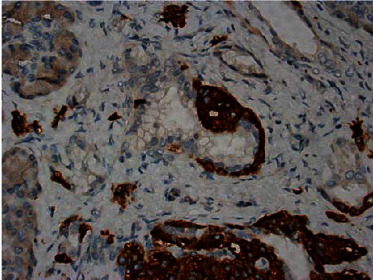
Ductuloinsular complexes at 400X with synaptophysin staining.

General surgery was consulted, and she subsequently underwent laparoscopic resection of the distal pancreas and splenectomy. A 0.6 × 0.6 × 0.3 cm pancreatic mass was identified (Figure 2). Pathological analysis staining with chromogranin and synaptophysin highlighted expanded islet cell nests with diffuse expansion of islets of Langerhans and ductal insular complexes consistent with nesidioblastosis (Figures 3–5). There was a focal region of increased density of pancreatic islet cells, which was associated with mild chronic pancreatitis and thought to potentially represent the region identified by increased signal intensity on PET imaging studies (Figure 1). No infiltrative malignant cell population, desmoplastic stromal reaction, or other features suggestive of carcinoma were identified.

The patient had a resolution of symptoms following the distal pancreatectomy and was safely discharged after the appropriate vaccination series for splenectomy.

## 3. Discussion

Our patient presented with severe symptomatic hypoglycemia resistant to diazoxide, requiring dextrose infusion with TPN. Her resistant hypoglycemia suggested insulinoma, but her biochemical analysis was not consistent with diagnosis. Her C-peptide and insulin levels were inappropriately normal, yet the proinsulin levels were not as elevated as expected. Routine imaging with CT suggested a possible lesion near the pancreas tail but overall was nondiagnostic. Endoscopic Ultrasound was unable to identify any lesion. A gallium-68 dotatate PET scan showed a focal radiotracer concentration in the distal pancreas suggesting a possible neuroendocrine tumor. She underwent laparoscopic resection of the distal pancreas mass, which confirmed nesidioblastosis [16]. In a similar case report, Patti et al. found hypoglycemic patients to have fasting hyperinsulinemia, while in our case, even though our patient presented with blood glucose levels in the 40s, her insulin and C-peptide levels on admission and repeat testing were not elevated from the normal range. More importantly, they were not suppressed, which would be expected with normal physiology [17]. Surreptitious sulfonylurea, insulin use, and malabsorption were excluded. Despite these findings, hyperinsulinemia was still suspected. Our patient was given diazoxide, an oral medication that prevents insulin secretion and is used to treat hyperinsulinism [18]. Despite the use of diazoxide, on hospital day 10, our patient had blood glucose levels in the 40s with nonsuppressed insulin and C-peptide levels, likely indicating increased base-level beta cell function and unregulated insulin secretion.

Diagnosing nesidioblastosis in adults is challenging because traditional imaging techniques lack the sensitivity to differentiate between focal and diffuse hyperinsulinism [7]. Common imaging modalities include computerized tomography, MRI, angiography, and transabdominal ultrasound [19–22]. Endoscopic ultrasound was found to have more than 90% accuracy in detecting lesions smaller than 3 cm, while intraoperative ultrasound was the most sensitive method for detecting tumors smaller than 1 cm [22]. This may not be feasible for patients with a small remnant gastric pouch. If imaging is negative, then an alternative method using arterial calcium stimulation with hepatic venous sampling (ASVS) can be done to localize areas of hyperfunctioning beta cells [17, 22]. If localization is detected, then a spleen-preserving distal pancreatectomy is recommended. ASVS can be technically cumbersome, and most cases have been identified via exploratory surgery [14, 17, 20, 23]. Histopathologic evaluation from the resected tissue is required to confirm the diagnosis.

In our literature review, previous cases used selective arterial calcium-stimulation tests to localize and guide partial pancreatectomy [14, 17]. We chose an alternative localization method. There are two other imaging modalities available. Neuroendocrine tumors uniquely express somatostatin receptors, allowing radiolabeled 111In-octreotide to be used in gamma imaging. Unfortunately, 111In-octreotide has a high radiation dose and is taken up physiologically, limiting the detection of smaller lesions [24]. Indium 111 isotope also provides a low image quality [24]. Next, the dotatate PET scan, which uses a radioactive 68-gallium labeled somatostatin analog, has shown to be useful and has a higher sensitivity in locating tumors/hyperplasia than an octreotide scan [25, 26]. The 68-gallium dotatate PET scan was able to localize the lesion in our patient, leading to successful resection and resolution of symptoms (Figure 1). Unfortunately, it is expensive, averaging $8,000 for this case, and the isotope is extremely limited.

Treatment of nesidioblastosis differs for adults and children. Isolate disease in children can present throughout the pancreas and can be cured with partial pancreatectomy of the focal lesion [8, 9]. The diffuse form in children necessitates near-total pancreatectomy [8]. The acquired form of nesidioblastosis most often occurs in adults following a Roux-en-Y bypass. In this setting, the lesions are more likely to be in the pancreatic tail and surgical resction with distal pancreatectomy has been a treatment for some patients [2, 8, 27, 28].

In many cases, surgery can confirm the diagnosis and provide treatment for severe cases [14, 17, 20, 23]. This contrasts with patients without evidence of increased beta islet cell mass. Vanderveen et al. surveyed 48 patients undergoing pancreatic resection for noninsulinoma pancreatogenous hypoglycemia in post-bariatric surgery patients. They found a significant rate of recurrent symptoms; only half of the patients were classified as highly/moderately surgically successful and 25% had no benefit [29]. In our case, distal pancreatectomy provided resolution of our patient's hypoglycemia and post-surgical pathology confirmed the diagnosis of nesidioblastosis.

## 4. Conclusion

We propose that with increasing rates of obesity and correlating gastric bypass surgeries, more providers will be faced with elucidating the various causes of postprandial and fasting hypoglycemia [13, 30]. We propose that with increased rates of weight loss surgery, hypoglycemic hyperinsulinemic nesidioblastosis will increase in prevalence. Differentiating the etiology can be challenging and requires a stepwise biochemical approach.

## Figures and Tables

**Table 1:** Biochemical evaluation for hypoglycemia.

Lab	Admission	HD#10^*∗*^	Reference
Glucose (mg/dL)	43	47	70–100
C-peptide (ng/mL)	2.3	3.1	0.8–3.5
Insulin, fasting (ulU/mL)	1.0	3.6	2.0–19.6
Proinsulin (pmol/L)	14.9	N/A	<18

^*∗*^Patient receiving diazoxide.

**Table 2:** Serologic exclusion of causes for hypoglycemia.

Lab	Value	Reference
Insulin autoantibody (U/mL)	<0.4	<0.4
Sulfonylurea screen	Negative	N/A
ACTH/cortrosyn stimulation (ug/dL)	1 hour cortisol 19.6	>20 ug/dL
Chromogranin A (ng/mL)	82	25–140
Transglutaminase IgG Abs (U/mL)	0.5	<1
Transglutaminase IgA Abs (U/mL)	0.5	<1
Gliadin peptide Ag IgG (U)	2	<20
Gliadin peptide Ag IgA (U)	3	<20

## Data Availability

Single case report with data are available upon request.
